# Uncoupling protein-2 attenuates glucose-stimulated insulin secretion in INS-1E insulinoma cells by lowering mitochondrial reactive oxygen species

**DOI:** 10.1016/j.freeradbiomed.2010.12.020

**Published:** 2011-03-01

**Authors:** Charles Affourtit, Martin Jastroch, Martin D. Brand

**Affiliations:** a Mitochondrial Biology Unit, Medical Research Council, Cambridge CB2 0XY, UK; b Buck Institute for Research on Aging, Novato, CA 94945, USA

**Keywords:** ∆ψ, mitochondrial membrane potential, DAPI, 4′,6-diamidino-2-phenylindole, DHE, hydroethidine, FCS, fetal calf serum, FCCP, carbonyl cyanide *p*-trifluoromethoxyphenylhydrazone, GSIS, glucose-stimulated insulin secretion, KRH, Hepes-buffered Krebs–Ringer medium, MitoSOX, mitochondria-targeted hydroethidine, MnTBAP, manganese tetrakis-(4-benzoic acid) porphyrin, MnTMPyP, manganese tetrakis-(*N*-methyl-4-pyridyl) porphyrin, ROS, reactive oxygen species, TTNPB, 4-[(*E*)-2-(5,6,7,8-tetrahydro-5,5,8,8-tetramethyl-2-napthalenyl)-1-propenyl]benzoic acid, UCP2, uncoupling protein-2, Pancreatic β cells, Glucose-stimulated insulin secretion, Uncoupling protein 2, Mitochondrial respiration, Reactive oxygen species, Coupling efficiency of oxidative phosphorylation, Type 2 diabetes, Metabolic syndrome, Free radicals

## Abstract

Glucose-stimulated insulin secretion (GSIS) by pancreatic β cells is regulated by mitochondrial uncoupling protein-2 (UCP2), but opposing phenotypes, GSIS improvement and impairment, have been reported for different *Ucp2*-ablated mouse models. By measuring mitochondrial bioenergetics in attached INS-1E insulinoma cells with and without UCP2, we show that UCP2 contributes to proton leak and attenuates glucose-induced rises in both respiratory activity and the coupling efficiency of oxidative phosphorylation. Strikingly, the GSIS improvement seen upon UCP2 knockdown in INS-1E cells is annulled completely by the cell-permeative antioxidant MnTMPyP. Consistent with this observation, UCP2 lowers mitochondrial reactive oxygen species at high glucose levels. We conclude that UCP2 plays both regulatory and protective roles in β cells by acutely lowering GSIS and chronically preventing oxidative stress. Our findings thus provide a mechanistic explanation for the apparently discrepant findings in the field.

Type 2 diabetes is a pandemic metabolic disorder that manifests by chronic hyperglycemia. Dysfunctional pancreatic β cells are involved prominently in the etiology of this disorder, as they contribute heavily to the impaired ability of diseased subjects to maintain glucose homeostasis. In healthy subjects, β cells respond to rising blood glucose levels by secreting insulin, which is a sign for peripheral tissues such as skeletal muscle and liver to take up or store glucose, respectively. When blood glucose levels rise, β cells increase their oxidative catabolism of glucose, which leads to an increased mitochondrial proton motive force and an increased cytoplasmic ATP/ADP ratio. In the canonical model of glucose-stimulated insulin secretion (GSIS)^2^, the boosted ATP/ADP poise closes plasma membrane K_ATP_ channels, causing plasma membrane depolarization, opening of voltage-sensitive calcium channels, calcium influx, and insulin secretion [1]. This order of events reflects an extraordinary bioenergetic design, as the β cell's response to glucose implies that its ATP/ADP ratio is controlled predominantly by ATP supply and not ATP demand, as is the case in most other cell types [2]. Moreover, the outlined scenario indicates that GSIS relies on the coupling efficiency of oxidative phosphorylation, as it seems essential that glucose oxidation and mitochondrial ATP synthesis are coupled tightly.

Pancreatic β cells contain a mitochondrial uncoupling protein, UCP2 [3]. The generally anticipated molecular function of this protein is partial dissipation of the proton motive force [4], which would (mildly) uncouple oxidative phosphorylation. UCP2 activity is thus expected to attenuate GSIS, a notion that is indeed supported by a growing body of experimental evidence (reviewed in [5]). However, it should be emphasized that a direct uncoupling function of UCP2 is not accepted universally [6,7] and, furthermore, that studies into the effects of UCP2 activity on GSIS are equivocal. Genetic knockout of *Ucp2* in mice was established originally on a mixed 129/SVJ × C57BL/6 background and resulted in greatly improved GSIS [3,8]. When this *Ucp2*-ablated strain was backcrossed onto three different genetic backgrounds, however, the GSIS phenotype was lost completely [8]. In fact, the resulting pure strains (C57BL/6 J, A/J, and 129/SvImJ) all exhibited *impeded* GSIS, which has been attributed to the chronic oxidative stress seen in these *Ucp2* knockout animals [8]. Similarly, the improved glucose tolerance reported by Zhang et al. [3] was not replicated in *Ucp2-*knockout backcrossed mice [9]. It is evident from these discrepant observations that unambiguous interpretation of studies with *Ucp2*-ablated mice is difficult given the confounding effects of genetic background. Such interpretation is complicated further by likely secondary or systemic effects caused by the ubiquitous and persistent absence of UCP2 protein from knockout animals. The inconsistent effect of *Ucp2* knockout on GSIS in different mouse strains, for example, may partly be due to the chronic lack of UCP2 in macrophages [10] and pro-opiomelanocortin neurons [11]. The ab initio absence of UCP2 in these tissues may well have differentially affected, respectively, the immune status and the hypothalamic glucose sensing ability of the various animal strains.

Interpretational difficulties of whole-animal studies can be avoided by using less complicated experimental systems such as clonal β cells. The relative strength of such a cellular approach is highlighted by our previous studies with INS-1E insulinoma cells [12–14], a widely used β-cell model that has retained most important characteristics of primary β cells, including GSIS [15]. We have shown that acute siRNA treatment of INS-1E cells can lead to 80–90% knockdown of UCP2 protein within 48 h of transfection [13,14]. This relatively acute knockdown of UCP2 protein causes a marked improvement in GSIS [13], which agrees well with the original *Ucp2* knockout studies in mice [3]. Our RNAi experiments furthermore revealed that UCP2 contributes significantly to the exceptionally high mitochondrial proton leak activity of INS-1E cells [13]. This finding led us to the assertion that UCP2 regulates the canonical GSIS pathway through modulating the coupling efficiency of oxidative phosphorylation [5,13,14], a notion that has indeed been put forward by many others (e.g., [3,16–18]). In light of the recent observation that hydrogen peroxide is an important noncanonical GSIS signal [19,20], however, we decided to explore the possibility that UCP2 activity affects GSIS by modulation of mitochondrial reactive oxygen species (ROS).

In this paper we report that the improvement of GSIS observed upon UCP2 knockdown in INS-1E cells is completely annulled in the presence of the cell-permeative antioxidant MnTMPyP. We show furthermore that UCP2 knockdown in *attached* INS-1E cells lowers mitochondrial respiratory activity, amplifies a glucose-induced increase in mitochondrial coupling efficiency, and, unexpectedly, improves the cells’ respiratory response to glucose. Importantly, we demonstrate that UCP2 activity lowers hydroethidine (DHE) oxidation at high glucose levels, but only when this ROS probe is targeted to mitochondria. We conclude that UCP2 lowers GSIS, at least partly, by lowering mitochondrial ROS.

## Experimental procedures

### Experimental system

INS-1E cells were obtained from Pierre Maechler and Claes Wollheim (Department of Internal Medicine, University Medical Center, Geneva, Switzerland) and grown as reported previously [15] in RPMI medium containing 11 mM glucose and 2 mM glutamine. An 80–90% knockdown of UCP2 protein was accomplished through siRNA transfection of INS-1E cells grown on XF24 (Seahorse Bioscience) or 96-well (Costar 3595; Corning) tissue culture plates. We have previously confirmed this level of UCP2 knockdown by Western analysis [14] and have also shown that such knockdown is achieved with siRNA oligonucleotides targeted at three different *Ucp2* exons [13]. Cells were grown overnight after seeding to about 50% confluence, transfected with 200 nM *Ucp*2-targeted siRNA (predesigned by Ambion, Huntingdon, UK; see [14]) in complex with 1.7 μg/ml Lipofectamine (Invitrogen, Paisley, UK), and subjected to GSIS, bioenergetic, and ROS assays 48 h posttransfection. To identify potential nonspecific effects, control cells were transfected in parallel with 200 nM scrambled siRNA (Silencer Negative Control 1; Ambion).

### GSIS

Cells seeded (at 40,000 cells per well) and transfected on 96-well plates were starved for 2 h in RPMI that lacked glucose and pyruvate and contained only 1% (v/v) fetal calf serum (FCS). Cells were washed twice with a glucose-free Hepes-buffered Krebs–Ringer medium composed of 135 mM NaCl, 3.6 mM KCl, 10 mM Hepes (pH 7.4), 0.5 mM MgCl_2_, 1.5 mM CaCl_2_, 0.5 mM NaH_2_PO_4_, 2 mM glutamine, and 0.1% (w/v) bovine serum albumin (KRH). Cells were then incubated in KRH ± 20 μM MnTMPyP (a cell-permeative superoxide dismutase mimetic [21]) for 30 min at 37 °C using a shaking plate incubator (Labnet International, Oakham, UK) set at 100 rpm. Note that metalloporphyrins such as MnTMPyP generally scavenge not only superoxide but, to different extents, also hydrogen peroxide, peroxynitrite, and lipid peroxyl radicals [21]. Next, the medium was replaced with KRH containing 2, 5, or 30 mM glucose, again in the presence or the absence of 20 μM MnTMPyP. After another 30-min shaking incubation at 37 °C, the medium was collected and centrifuged to pellet any detached cells. Supernatants were assayed for insulin by ELISA (Mercodia, Uppsala, Sweden) using mouse insulin as a standard. Note that our KRH formulation contains 2 mM glutamine to maintain UCP2 protein levels in control cells (cf. [22]), but lacks the usually included sodium bicarbonate to prevent alkalinization of the medium during the assay.

### Mitochondrial bioenergetics

In experiments designed to measure bioenergetic parameters under conditions identical to those applied during GSIS assays (Figs. 2 and 3), cells were seeded (40,000 cells/well) and transfected on Seahorse XF24 plates. On the day of assay, the cells were starved for 2 h in RPMI that lacked glucose and pyruvate and contained only 1% (v/v) FCS. The cells were washed twice with glucose-free KRH and incubated in this medium ± 20 μM MnTMPyP for 10 min in a 37 °C air incubator. The XF24 plate was then transferred to a temperature-controlled (37 °C) Seahorse analyzer and subjected to a 10-min equilibration period and 3 assay cycles, comprising a 1-min mix, 2-min wait, and 3-min measure period each (cf. [14]); then glucose (2, 5, or 30 mM in KRH) was added by automatic pneumatic injection. After 10 further assay cycles, oligomycin (1 μg/ml) was added to inhibit the ATP synthase and thus approximate the proportion of respiration used to drive ATP synthesis (coupling efficiency). Each experimental trace was ended by addition of a rotenone (1 μM) and myxothiazol (2 μM) mixture to determine the nonmitochondrial respiratory rate, which was subtracted from all other rates. The stated inhibitor concentrations gave maximum effects as verified by doubling the concentrations. Coupling efficiency was calculated as the oligomycin-sensitive fraction of glucose-stimulated mitochondrial respiratory activity (last rate before oligomycin addition). The mitochondrial respiratory response to glucose was normalized as the ratio of glucose-stimulated and basal oxygen uptake activities (last rates before oligomycin and glucose addition, respectively).

**Fig. 1:**
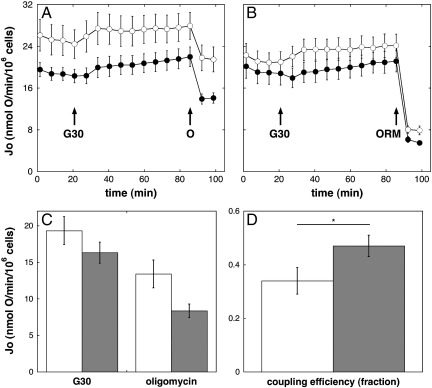
UCP2 knockdown lowers mitochondrial respiratory rate and increases the coupling efficiency of attached INS-1E cells. (A and B) Time-resolved absolute respiratory rates (*J*_o_), normalized to cell number as measured by nuclear staining, of cells transfected with *Ucp2*-targeted (closed symbols) or scrambled siRNA (open symbols) and treated as described under Experimental procedures. After four Seahorse assay cycles at 2 mM glucose, cells were subjected to 30 mM glucose (G30) and, in parallel experiments, either oligomycin (O) or a mixture of oligomycin, rotenone, and myxothiazol (ORM). Each time point represents the average ± SEM (*n* = 8–12) of 8–12 separate wells sampled from seven independent Seahorse XF24 plates. (C) Respiratory rates in the presence of the oligomycin/rotenone/myxothiazol mix were averaged and subtracted from all other rates to yield mitochondria-specific respiratory activities. Glucose-stimulated activity (G30) is the average of the last two rate measurements before oligomycin addition, and the oligomycin-insensitive activity (oligomycin) is the average of the two rates after this addition. (D) Coupling efficiencies were calculated as the fraction of glucose-stimulated mitochondrial oxygen uptake that is sensitive to oligomycin and thus reflects respiratory activity used to drive ATP synthesis. The data in (C) and (D) are averages ± SEM (*n* = 13–15) of 13 (scrambled siRNA, open bars) or 15 (*Ucp2*-targeted siRNA, shaded bars) separate wells sampled from seven independent XF24 plates. *p < 0.05.

In experiments designed to determine absolute oxygen consumption rates (Fig. 1), cells were also seeded (20,000 cells/well) and transfected on XF24 plates. On the day of assay (i.e., 48 h posttransfection), however, the cells were not starved of glucose in RPMI, but incubated instead for 1 h in a 37 °C air incubator in KRH containing 2 mM glucose but lacking bovine serum albumin. After a 10-min equilibration in the Seahorse analyzer and 4 assay cycles to measure basal respiration, the cells were subjected to 30 mM glucose. In parallel experiments, either oligomycin (1 μg/ml) or a mixture of oligomycin (1 μg/ml), rotenone (1 μM), and myxothiazol (2 μM) was added after 10 further cycles to allow assessment of coupling efficiency and nonmitochondrial respiratory activity, respectively (see the Fig. 1 legend for more detail). Immediately after the Seahorse assay, the cells were fixed with 4% (w/v) paraformaldehyde, and nuclear DNA was then stained with 4′,6-diamidino-2-phenylindole (DAPI; applied at a 1:5000 dilution). DAPI-stained cells were imaged using a wide-field microscope (10× magnification) and view fields were assembled to construct images of individual XF24 wells. Cells (nuclei) were counted using Image Analyst software (http://www.imageanalyst.net) and specific oxygen consumption rates were calculated as nanomoles atomic oxygen consumed per minute per 10^6^ cells.

### ROS production

Cells seeded (40,000 cells/well) and transfected on 96-well plates were starved for 2 h in RPMI that lacked glucose and pyruvate and contained only 1% (v/v) FCS. The cells were washed twice with glucose-free KRH and incubated in this medium ± 20 μM MnTMPyP for 30 min at 37 °C using a shaking plate incubator (Labnet International) set at 100 rpm. After these two glucose-starvation periods that were identical to the experimental GSIS conditions, the medium was replaced with KRH containing 2, 5, or 30 mM glucose, again in the presence or the absence of 20 μM MnTMPyP, and either 5 μM MitoSOX (Invitrogen M36008) or 100 μM DHE (Invitrogen D11347) was added. Plates were transferred immediately to a spectrofluorimetric microplate reader (Molecular Devices Spectramax Gemini XPS) and fluorescence was recorded without mixing at 28-s intervals for 1 h. Fluorescent oxidation products were excited at 510 nm and light emission was recorded at 580 nM (MitoSOX) or 610 nm (DHE).

### Statistics

Statistical analysis was performed with GraphPad Prism version 5.0a for Mac OS X (GraphPad Software, San Diego, CA, USA; www.graphpad.com). All mean differences between experimental systems and conditions were evaluated by one-way ANOVA with Tukey's posttest, except for the difference shown in Fig. 1D, which was assessed by an unpaired *t* test. The level of statistical significance is indicated in the figures by asterisks.

## Results

We have reported previously that knockdown of UCP2 by RNAi leads to improved GSIS in INS-1E insulinoma cells [13]. To assess the bioenergetics of INS-1E cells under conditions identical to those applied during the GSIS experiments, we used a Seahorse extracellular flux analyzer (cf. [14]) that enables noninvasive, real-time determination of mitochondrial respiratory activity and coupling efficiency in *attached* cells. This approach is superior to our previous bioenergetic assessment of *trypsinized* INS-1E cells [13] as it removes the concern that the UCP2 knockdown phenotype is facilitated by a trypsin-induced stress response.

### UCP2 knockdown lowers mitochondrial respiratory activity of attached INS-1E cells

**Fig. 2:**
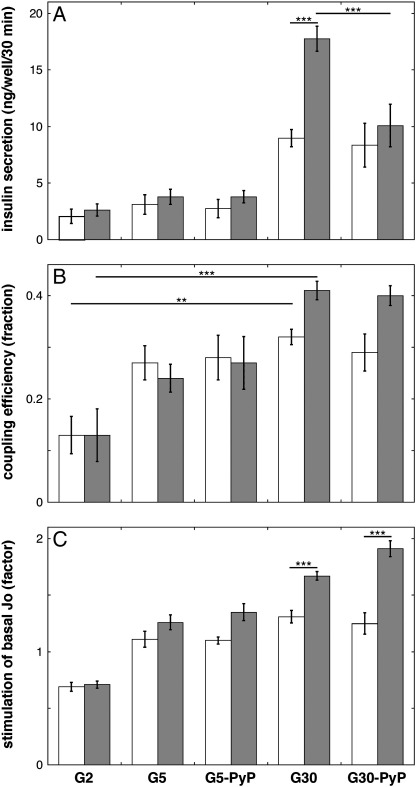
UCP2 knockdown improves GSIS in a MnTMPyP-sensitive manner, amplifies a glucose-induced rise in coupling efficiency, and improves the cells’ respiratory response to glucose. Cells transfected with *Ucp2*-targeted or scrambled siRNA (shaded and open bars, respectively) were first starved of glucose as described under Experimental procedures and then subjected to glucose in the absence (G2, G5, G30) or presence (G5-PyP, G30-PyP) of 20 μM MnTMPyP; G2, G5, and G30 reflect 2, 5, and 30 mM glucose, respectively. (A) Insulin secretion data are averages ± SEM of four independent experiments with each condition assayed six times. (B) Mitochondrial coupling efficiencies and (C) the respiratory responses of the cells to glucose are averages ± SEM of 4–11 separate XF24 runs. These bioenergetic parameters were calculated from Seahorse traces as described under Experimental procedures. **p < 0.01 and ***p < 0.001.

Figs. 1A and 1B present time-resolved absolute respiratory activities exhibited by attached INS-1E cells that were transfected with scrambled or *Ucp2*-targeted siRNA and that had been starved for 1 h before assay of respiration in KRH containing merely 2 mM glucose and lacking bovine serum albumin. After four basal rate measurements, the glucose concentration was raised to 30 mM and, in parallel experiments, the ATP synthase (Fig. 1A) and also the mitochondrial respiratory chain (Fig. 1B) were subsequently inhibited, respectively, by oligomycin or a mixture of oligomycin, rotenone, and myxothiazol. Figs. 1A and 1B show that a raised glucose concentration causes small respiratory increases in cells with and without UCP2. Although these increases are relatively modest and the respiratory rates are somewhat variable, it may be noted that UCP2-containing cells achieve, relatively rapidly, an activity that is steadier than that exhibited by UCP2-lacking cells. In fact, glucose-stimulated cells without UCP2 do *not* reach a steady respiratory rate until other effectors are added (cf. the normalized respiratory responses to glucose shown in Fig. 2C). As expected, oxygen uptake is lowered both by oligomycin (Fig. 1A) and by the mix of oligomycin, rotenone, and myxothiazol (Fig. 1B). Under all conditions and at all time points, the absolute total respiratory activity of UCP2-containing cells is higher than that of their UCP2-ablated counterparts. Fig. 1C shows these respiratory activities corrected for nonmitochondrial oxygen consumption and, although statistically not significant, it can be seen that UCP2 knockdown tends to lower mitochondrial respiration at 30 mM glucose both in the absence and in the presence of oligomycin. This finding is consistent with our previous study with trypsinized INS-1E cells [13] and indicates that UCP2 activity stimulates mitochondrial proton leak rate in attached INS-1E cells. The observation that nonmitochondrial respiration is decreased by UCP2 knockdown as well (Fig. 1B) is again consistent with the trypsinized cell data, although the nature of this effect remains unclear. The absolute mitochondrial respiratory activities shown in Fig. 1C are about 70–80% higher than the “trypsinized” activities [13], which is most probably due to a different experimental design in terms of assay medium composition and glucose concentration. The rate-lowering effect of UCP2 knockdown is more pronounced in the presence than in the absence of oligomycin (Fig. 1C), which is reflected by the comparatively high coupling efficiency of UCP2-depleted cells (Fig. 1D). Note that the effect of UCP2 knockdown on coupling efficiency (a normalized parameter) is indeed statistically significant.

We stress that we define coupling efficiency as the proportion of mitochondrial respiration that is used to drive ATP synthesis, i.e., the respiratory fraction that is sensitive to oligomycin. In principle, therefore, an increased coupling efficiency could be due to either decreased proton leak rate or increased rate of ADP phosphorylation. A lowered proton leak rate would *decrease* respiration, whereas a rise in phosphorylation rate would *increase* it. UCP2 knockdown results in both an increased coupling efficiency (Fig. 1D) and a decreased mitochondrial respiratory activity (Fig. 1C), demonstrating that knockdown of UCP2 is associated with decreased proton leak rate across the mitochondrial inner membrane.

### UCP2 dampens a glucose-induced increase in the coupling efficiency of INS-1E cells

Next, we probed coupling efficiencies in attached cells under conditions that were *identical* to those applied during the GSIS assay. First, we performed a static GSIS experiment challenging glucose-starved INS-1E cells with and without UCP2 with 2, 5, and 30 mM glucose. The GSIS data shown in Fig. 2A reaffirm that acute UCP2 knockdown increases insulin secretion significantly at 30 mM glucose, whereas it does not affect insulin release at low glucose levels. This GSIS improvement is somewhat more pronounced than reported previously [13], which is probably due to the presence of glutamine in all our current buffers to ensure that UCP2 protein is retained in the control cells throughout the assay [22]. In parallel experiments, we then determined the effects of glucose challenge on the mitochondrial bioenergetics of starved INS-1E cells with and without UCP2. Fig. 2B shows that a rise in glucose concentration from 2 to 30 mM increases the coupling efficiency of UCP2-containing cells from 0.13 to 0.32 on average. This increase is more significant in UCP2-depleted cells, leading to a coupling efficiency of 0.41 on average at 30 mM glucose. It can be taken from these data that UCP2 activity dampens a glucose-induced increase in mitochondrial coupling efficiency. This outcome is consistent with our previous conclusion that UCP2 attenuates GSIS because of its considerable contribution to mitochondrial proton leak [13].

### UCP2 lowers the respiratory response of INS-1E cells to glucose

Figs. 1A and 1B show that the absolute respiratory activity of INS-1E cells is increased modestly when the glucose concentration is raised from 2 to 30 mM. The data shown in Fig. 2C were obtained in a different set of experiments and quantify this respiratory effect by normalizing glucose-stimulated activity to the basal activity measured in glucose-starved INS-1E cells with and without UCP2. When glucose levels are kept low after the basal “starved” measurements (i.e., when KRH with only 2 mM glucose is added), respiration in cells with and without UCP2 declines gradually, leading to rates after 10 assay cycles that are approximately 70% of the initial activity (Fig. 2C, G2). This respiratory decline may reflect the cells’ response to the prolonged glucose deprivation. When UCP2-containing cells are challenged with 5 or 30 mM glucose, the respiratory activity increases to values that are, respectively, about 10 and 30% higher than the basal activity (Fig. 2C, G5 and G30, open bars). Knockdown of UCP2 improves this normalized respiratory response, at both 5 and 30 mM glucose, which leads to stimulated rates that are, respectively, about 25 and 70% above the basal rate (Fig. 2C, G5 and G30, shaded bars). The improvement at 30 mM glucose is statistically significant and it can thus be concluded that, rather unexpectedly, UCP2 restrains a glucose-induced increase in mitochondrial respiration. When the data shown in Figs. 1A and 1B are normalized to basal mitochondrial respiration, a similar, though somewhat less pronounced, UCP2 phenotype transpires (not shown). Variation in the size of the UCP2 effect is probably due to differences in experimental design, particularly with respect to glucose starvation before the respective sets of measurements (see Experimental procedures).

### GSIS improvement upon UCP2 knockdown is annulled when ROS are scavenged

In light of increasing evidence that ROS are important GSIS signals [19,20], we explored the possibility that GSIS regulation by UCP2 is affected not only by coupling efficiency but also by mitochondrial ROS. To do this, we repeated the GSIS and bioenergetic experiments described in the previous two paragraphs, but now in the presence of MnTMPyP, a potent and cell-permeative antioxidant [21]. Strikingly, the UCP2 knockdown-mediated rise in insulin secretion seen at 30 mM glucose was fully annulled when ROS were scavenged by 20 μM MnTMPyP (Fig. 2A). This observation associates UCP2 with ROS in terms of GSIS regulation, but not in the manner suggested by Krauss et al. [23], who reported a dose-dependent MnTBAP-induced stimulation of insulin secretion by pancreatic islets. Because the effect of MnTBAP, an antioxidant slightly less potent than MnTMPyP [21], was observed exclusively in UCP2-containing islets, it was interpreted as prevention of superoxide-mediated UCP2 activation [23]. Quite differently, MnTMPyP does not affect GSIS by UCP2-containing INS-1E cells (Fig. 2A). Instead, our data reveal that a lack of UCP2 protein can be compensated for by the presence of a ROS scavenger and therefore suggest that UCP2 activity attenuates GSIS, at least in part, by dampening ROS levels.

Importantly, the presence of 20 μM MnTMPyP during respiration measurements with glucose-starved INS-1E cells has no effect on either coupling efficiency (Fig. 2B) or normalized glucose-stimulated respiration (Fig. 2C). The absolute value of these bioenergetic parameters, in cells both with and without UCP2, is not significantly affected by ROS scavenging. The MnTMPyP insensitivity of the UCP2-knockdown effect on the INS-1E coupling efficiency is somewhat surprising, because the superoxide activation of UCP2-mediated effects in pancreatic islets [23] had raised the expectation that MnTMPyP would increase coupling efficiency of UCP2-containing cells at 30 mM glucose.

### MitoSOX oxidation by INS-1E cells

**Fig. 3:**
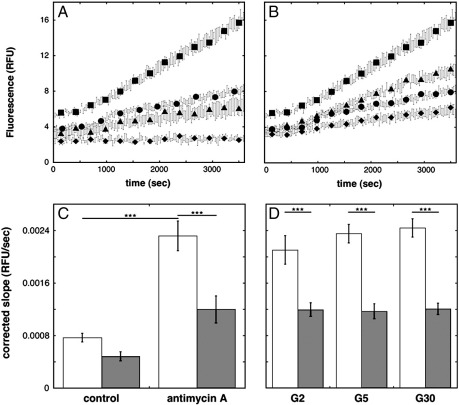
MnTMPyP lowers the rate of MitoSOX oxidation by INS-1E cells. (A and B) Typical time-resolved fluorescence observed upon the oxidation of 5 μM MitoSOX by nontransfected INS-1E cells that were *not* subjected to periods of glucose starvation. Traces are shown to illustrate assays that were done without any effector (circles) or added probe (A, diamonds) and assays that were performed in the presence of 15 μM antimycin A (squares), 15 μM FCCP (A, triangles), 20 μM MnTMPyP (B, diamonds), or 15 μM antimycin A and 20 μM MnTMPyP (B, triangles). Data are averages ± SEM of 4–6 wells sampled from a single 96-well plate and, to aid clarity, symbols are shown only for every 10th measurement. (C) MitoSOX oxidation rates in the presence (shaded bars) and the absence (open bars) of 20 μM MnTMPyP were calculated from the slopes (after the first 1000 s) of progress curves exemplified in (A) and (B). Data are averages ± SEM of 34–38 wells sampled from seven plates and 18–22 wells sampled from four plates in the absence and the presence of MnTMPyP, respectively. (D) MitoSOX oxidation by nontransfected glucose-starved INS-1E cells (see Experimental procedures) subjected to 2, 5, and 30 mM glucose (G2, G5, and G30, respectively) in the presence (shaded bars) and the absence (open bars) of 20 μM MnTMPyP. Data are averages ± SEM of 22–24 wells sampled from three plates. RFU, relative fluorescence units. ***p < 0.001.

Although the MnTMPyP effect on GSIS in itself shows that this chemical is active when added to INS-1E cells, we wished to establish more directly that it scavenged ROS. If MnTMPyP indeed scavenges ROS (including superoxide) in INS-1E cells, then it should lower the rate of DHE oxidation, an activity that can be monitored fluorimetrically and that is widely used to detect superoxide in cultured cells [24]. Note that DHE is oxidized not only by superoxide but also by hydrogen peroxide (in the presence of peroxidases) and intracellular oxidoreductases [24]. Fig. 3A shows several typical fluorescence progress curves that reflect the oxidation by INS-1E cells of MitoSOX, a DHE derivative that is targeted to mitochondria because of its conjugation to the lipophilic triphenylphosphonium cation. The slope of these fluorescence traces reports mitochondrial ROS [24], a notion that is corroborated by the expected responses of MitoSOX oxidation to antimycin A and the uncoupling agent FCCP, which increase and decrease this slope, respectively (Fig. 3A). Similar traces shown in Fig. 3B illustrate qualitatively that MnTMPyP lowers the rate of MitoSOX oxidation both in the absence and in the presence of antimycin A. This effect is statistically significant only in the presence of antimycin A (Fig. 3C), but demonstrates that MnTMPyP effectively lowers ROS concentrations in the presence of MitoSOX. From this it may be inferred that MnTMPyP indeed lowers (mitochondrial) ROS levels in INS-1E cells. Furthermore, Fig. 3D shows that MnTMPyP decreases MitoSOX oxidation rates at low and high glucose levels when nontransfected INS-1E cells are subjected to conditions applied during the GSIS experiments. Raising the glucose concentration from 2 to 30 mM may increase ROS concentration slightly, but this effect is statistically not significant (Fig. 3D). It is clear from these data, however, that the MitoSOX assay is well suited to assessing if and how UCP2 knockdown affects mitochondrial ROS under GSIS-like conditions.

### UCP2 knockdown effects on DHE oxidation

**Fig. 4:**
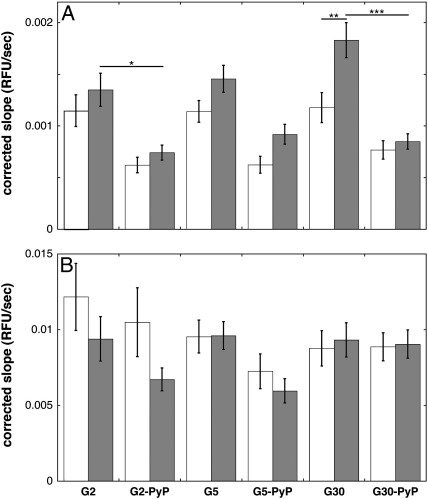
UCP2 activity lowers mitochondrial ROS at high glucose levels. Cells transfected with *Ucp2*-targeted (shaded bars) or scrambled (open bars) siRNA were first starved of glucose as described under Experimental procedures and then subjected to glucose in the absence (G2, G5, G30) or presence (G2-PyP, G5-PyP, G30-PyP) of 20 μM MnTMPyP; G2, G5, and G30 reflect 2, 5, and 30 mM glucose, respectively. (A) MitoSOX (5 μM) oxidation rates were obtained as described for Fig. 3 and (B) DHE (100 μM) oxidation rates were calculated from the slopes of the first 500 s of the fluorescence traces. Data are averages ± SEM of 25–30 wells sampled from six 96-well plates. *p < 0.05, **p < 0.01 and ***p < 0.001.

Fig. 4A shows that MitoSOX oxidation by transfected UCP2-containing cells (scrambled control) does not change discernibly when the cells are subjected to increasing glucose concentrations. UCP2 knockdown results in a statistically significant rise in the MitoSOX oxidation rate at 30 mM glucose, which effectively means that this rate tends to exhibit a glucose dependence in UCP2-ablated cells. In other words, the data suggest that UCP2 activity prevents a glucose-induced increase in mitochondrial ROS. Importantly, the UCP2 knockdown-mediated increase in MitoSOX oxidation at 30 mM glucose is fully abolished in the presence of MnTMPyP (Fig. 4A).

Unlike MitoSOX, DHE itself is not targeted to mitochondria. The oxidation rate of DHE is unaffected by glucose concentration, in cells both with and without UCP2 (Fig. 4B). Moreover, MnTMPyP does not significantly affect DHE oxidation at any glucose level, in cells neither with nor without UCP2 (Fig. 4B).

## Discussion

The possibility that UCP2 might be a relevant GSIS regulator has provoked considerable interest (see, e.g., [5,25–27] for reviews) as this mitochondrial carrier could well be an attractive therapeutic target in the treatment of type 2 diabetes. The various genetically modified mouse models that have been established to explore this possibility, however, exhibit inconsistent phenotypes: glucose tolerance and GSIS in the original *Ucp2*-deficient mouse are improved [3], whereas in more backcrossed strains they are unaffected [9] or impeded [8]. Not surprisingly, these diametrically opposite observations have led to rather different suggestions for the in vivo function of UCP2. On one hand, a pathological role in the etiology of type 2 diabetes has been suggested [26], and on the other, a physiological role in the protection against oxidative stress has been proposed [27]. If UCP2 is to be considered a therapeutic target in type 2 diabetes, it seems essential to shed light on these disparate findings. Our data increase the understanding as to how GSIS is regulated by UCP2 and thus provide mechanistic grounding for the apparently discrepant findings in the field. Because all our data were obtained using a clonal β-cell model, obvious caution is warranted in their physiological interpretation.

### GSIS regulation by UCP2

Arguably our most striking observation is that GSIS improvement after UCP2 knockdown is abolished by MnTMPyP (Fig. 2A), which indicates that UCP2 attenuates GSIS by depressing ROS levels. This result is consistent with the recent awareness that ROS are an important signal during GSIS [19,20]. Pi and colleagues showed that addition of 1–4 μM H_2_O_2_ to mouse islets or insulinoma cells increases insulin secretion at low glucose levels. Insulin secretion is also increased by diethyl maleate, a compound that raises intracellular H_2_O_2_ levels [20]. GSIS is inhibited by H_2_O_2_ scavengers, but is unaffected by a cell-permeative superoxide dismutase [20]. In agreement with these data, our UCP2-containing cells exhibit GSIS that is insensitive to MnTMPyP (Fig. 2A). However, the MnTMPyP sensitivity of UCP2-depleted cells indicates that H_2_O_2_ is not a relevant signal during the GSIS that is facilitated by a lack of UCP2. ROS-mediated GSIS attenuation by UCP2, therefore, probably occurs via a mechanism (probably involving superoxide) different from the one that accounts for the findings of Pi et al. [20].

INS-1E cells tend to exhibit glucose-stimulated MitoSOX oxidation, but only in the absence of UCP2 (Fig. 4A). Importantly, oxidation of DHE *not* targeted to mitochondria is fully glucose insensitive even in the absence of UCP2 (Fig. 4B). Moreover, MitoSOX oxidation is decreased by MnTMPyP (Fig. 4A), which annuls the UCP2-dependent difference seen at high glucose. Together, these data suggest that UCP2 lowers GSIS by dampening glucose-induced ROS production. Two points of caution, however, deserve attention.

First, MitoSOX equilibrates across the mitochondrial inner membrane according to the Nernst equation and thus accumulates in a membrane potential (∆ψ)-dependent manner [24]. It is therefore conceivable that the MitoSOX concentration achieved in mitochondria lacking UCP2 is higher than that reached in their UCP2-containing counterparts, based on the assertion that UCP2 activity partially dissipates the ∆ψ. Because MitoSOX oxidation depends on probe concentration (the dose dependency is linear up to 50 nM in cultured neurons [28]), the UCP2 effect at 30 mM glucose (Fig. 4A) could formally be due to a probe accumulation difference. This eventuality seems unlikely, however, because we applied a saturating MitoSOX concentration (5 μM). More importantly, UCP2 knockdown is not expected to increase the ∆ψ exclusively at 30 mM glucose, but also (perhaps even *more* so given the underlying kinetics of oxidative phosphorylation [14]) at 2 and 5 mM glucose. MitoSOX oxidation at these glucose levels is not affected significantly by UCP2 knockdown (Fig. 4A).

The second issue that requires attention is the apparent lack of effect of UCP2 knockdown on DHE oxidation (Fig. 4B). Even without a specific targeting moiety, DHE is expected to achieve concentrations that are sufficient to detect mitochondrial ROS. It is important to appreciate in this respect that pancreatic β cells contain NADPH oxidases that produce considerable amounts of cytoplasmic superoxide [29]. It should be realized too that DHE is oxidized not only by superoxide but also by H_2_O_2_ (in the presence of peroxidases) and intracellular oxidoreductases [24], which probably explains the relative insensitivity of DHE oxidation to MnTMPyP (Fig. 4B). We assert that DHE is mainly oxidized by cytoplasmic INS-1E events (supported by insensitivity of DHE oxidation to antimycin A; data not shown) that mask any effect UCP2 may have on mitochondrial ROS production. MitoSOX oxidation, on the other hand, is dominated by mitochondrial ROS because of the vast accumulation of this probe into the mitochondrial matrix, which is evidenced by stimulatory and inhibitory effects of antimycin A and FCCP, respectively (Fig. 3A).

The differential effect of UCP2 on MitoSOX and DHE oxidation (Fig. 4) is readily interpreted as an exclusive effect of UCP2 on mitochondrial ROS production. Our data do not explain *how* these ROS stimulate GSIS. Presumably it is attenuation of the proton motive force by UCP2 that lowers mitochondrial ROS production (Fig. 4) and decreases the coupling efficiency of oxidative phosphorylation (Fig. 2B). Decreased coupling efficiency attenuates glucose-induced rises in the cytoplasmic ATP/ADP ratio. Further research is required to quantify the relative importance of the UCP2 effects on ROS and ATP/ADP in terms of GSIS regulation, although the full sensitivity to MnTMPyP (Fig. 2A) suggests strongly that the UCP2 phenotype is mediated mainly via ROS in our experiments. Interestingly, our bioenergetic data disclose another novel mechanism by which mitochondrial uncoupling may lower GSIS, as UCP2 activity also dampens the cells’ respiratory response to glucose (Fig. 2C). It is currently unclear how the self-reinforcement of glucose oxidation is effected, but ROS, ATP/ADP, or Krebs cycle intermediates are all possible signals. The MnTMPyP insensitivity of the UCP2 effect (Fig. 2C), however, implies that mitochondrial ROS are the least likely cue. The relative tardiness of the effect (Figs. 1A and 1B) indicates that mitochondrial biogenesis may be required.

### UCP2 function and regulation

Although the attenuating effect of UCP2 activity on glucose-stimulated coupling efficiency (Fig. 2B) and mitochondrial ROS production (Fig. 4) agrees with a primary proton leak function, it is possible that a different UCP2 activity accounts for these phenotypes. For example, putative pyruvate export by UCP2 [30] could explain in principle how UCP2 dampens glucose-dependent respiration, ROS production, and coupling efficiency, by oxidizing and slowing the electron transport chain. However, measurements of absolute respiratory activity in cells with and without UCP2 distinguish simply between a proton leak and pyruvate export function. As demonstrated before in pancreatic islets [31] and trypsinized INS-1E cells [13], UCP2 knockdown *lowers* the mitochondrial respiratory rates of attached INS-1E cells, in the absence but particularly the presence of oligomycin (Fig. 1C). This finding demonstrates that UCP2 contributes to mitochondrial proton leak. If UCP2 exported pyruvate, then the absolute respiratory activities would have *increased* upon UCP2 knockdown.

Previous work from our laboratory has led to the notion that UCPs attenuate ∆ψ only when they are activated, for example, by superoxide [32]. Although functional regulation of novel UCPs is the subject of ongoing debate [6,7,33], some experimental evidence supports UCP2 activation in cells. In thymocytes, for example, UCP2 activity is stimulated by the retinoic acid analogue TTNPB [34] and all UCP2-mediated pathological effects in pancreatic islets depend on superoxide [23]. However, our current data do not favor UCP2 activation in INS-1E cells by superoxide. ROS scavenging by MnTMPyP in UCP2-containing INS-1E cells does *not* lead to improved GSIS (Fig. 2A), coupling efficiency (Fig. 2B), or an increased normalized respiratory response to glucose (Fig. 2C). Although MnTMPyP clearly lowers mitochondrial ROS (Figs. 3B, 3C, 3D, and 4A), it remains possible that the concentration we applied (20 μM) is insufficient to acutely reverse already activated UCP2. In that case, it would be surprising that 10–20 μM MnTBAP prevents UCP2 activation in pancreatic islets [23]. Similarly, INS-1E respiration is not affected significantly by TTNPB either, even when the compound is added in the presence of oligomycin (C. Affourtit and M.D. Brand, unpublished data). In our hands, TTNPB *does* in fact inhibit GSIS, but in a UCP2-independent manner. Together with the recent observation that β-cell UCP2 is not activated by fatty acids [35], our data may suggest that UCP2 activity is not regulated acutely in cultured β cells, perhaps because the protein is permanently or constitutively activated. In that case, activity in these cells would be controlled exclusively by transcription and translation against a background of exceptionally rapid UCP2 protein degradation [22].

## Conclusion

The work presented in this paper highlights the relative strength of the INS-1E model, providing new insights into UCP2 function and regulation. Importantly, we have shown that UCP2 attenuates GSIS in a manner that can be mimicked by the antioxidant MnTMPyP. This finding reaffirms that UCP2 is an acute regulator of GSIS, which agrees with the GSIS phenotype exhibited by the original *Ucp2*-knockout mouse [3]. Moreover, we have shown that UCP2 lowers mitochondrial ROS in INS-1E cells at high glucose concentrations. This particular result provides a direct mechanistic basis for the oxidative stress phenotype exhibited by the more recently established *Ucp2*-deficient mouse strains [8]. We conclude that through modulation of mitochondrial ROS production, UCP2 plays both regulatory and protective roles in pancreatic β cells as its activity will attenuate GSIS acutely and, in the long term, will also prevent oxidative stress.
